# Surgical Challenges and Outcomes in Treating a Floating Upper Arm With Multiple Humerus Fractures and Radial Neck Fractures

**DOI:** 10.7759/cureus.66468

**Published:** 2024-08-08

**Authors:** Nikolaos P Sachinis, Nikolaos Mitsios, Maria Baxevanou, Christos Koukos, Alexandros Givissis, Panagiotis Givissis

**Affiliations:** 1 First Orthopaedic Department, "Georgios Papanikolaou" Hospital, Aristotle University of Thessaloniki, Thessaloniki, GRC; 2 Sports Trauma and Pain Institute, "Georgios Papanikolaou" Hospital, Aristotle University of Thessaloniki, Thessaloniki, GRC; 3 Orthopaedic Department, School of Medicine, European University of Cyprus, Thessaloniki, GRC

**Keywords:** floating elbow variant, supracondylar humeral fracture, polytrauma patient, radial neck fracture, humerus, comminuted fracture

## Abstract

Complex humerus fractures associated with high-energy trauma present significant surgical challenges due to their impact on limb functionality and structural integrity. This case report details the treatment of a floating upper arm injury, characterized by multiple humerus fractures and a radial neck fracture, in a 50-year-old male following a vehicular accident. The patient exhibited fractures at the proximal, mid-shaft, and distal segments of the humerus, necessitating an integrated surgical approach to effectively manage these injuries. Dual surgical approaches and perpendicular plating were employed to address the multifaceted nature of the fractures. The patient achieved satisfactory functional recovery, regaining a significant range of motion, which highlights the efficacy of the chosen surgical strategy. This case contributes to the existing literature by illustrating the benefits of specific surgical techniques in managing complex humerus fractures, emphasizing the necessity for meticulous planning and execution to optimize patient outcomes.

## Introduction

The management of complex humeral fractures, especially those involving multiple segments, poses significant challenges due to the intricate anatomy and mechanical demands of the upper limb. These fractures frequently result from high-energy trauma, necessitating a multidisciplinary approach to ensure optimal outcomes and minimize complications [1]. The proximity of vital neurovascular structures to the humerus requires precise surgical interventions to avoid iatrogenic injury [2].

Given the complexity of such injuries, the floating elbow concept, involving simultaneous humeral and forearm fractures, stresses the necessity for a holistic yet targeted approach to fracture management. This ensures not only the stabilization of the fracture but also the preservation of limb functionality [3]. Effective management relies heavily on advanced fixation techniques that adapt to the biomechanical complexities of the arm and the specific requirements of each fracture segment [4].

This case report aims to detail the surgical strategy employed for the treatment of a patient with multiple humeral fractures and a radial neck fracture, focusing on the technical considerations and the efficacy of perpendicular plating techniques in providing stable fixation and facilitating bone healing.

## Case presentation

**Figure 1:**
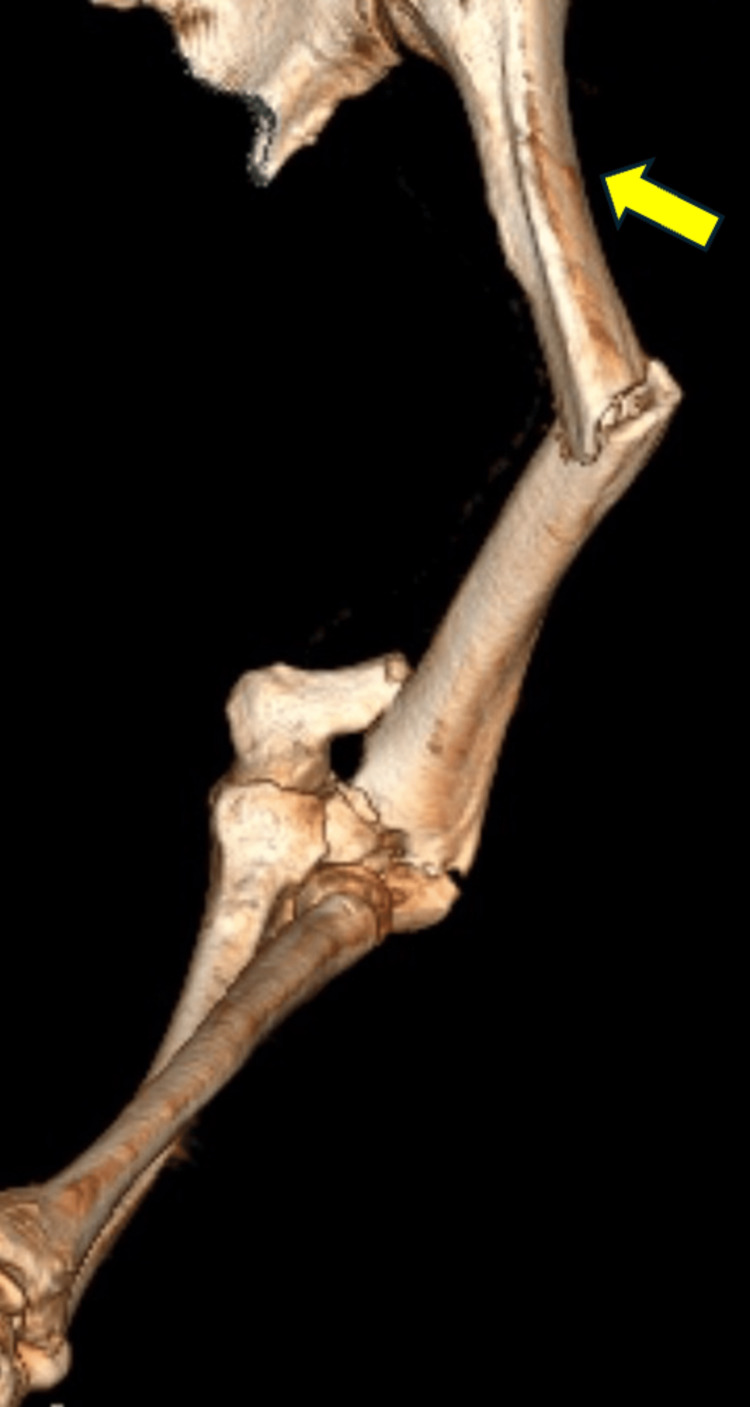
3D reconstruction of the CT scan depicting the radial head fracture and the supra-intercondylar, midshaft, and proximal incomplete humeral fractures. The incomplete fracture is shown with a yellow arrow.

A 50-year-old man was admitted to the Emergency Department of our hospital following a car accident, presenting with peritraumatic amnesia and a Glasgow Coma Scale score of 15/15. He exhibited injuries to his left elbow and upper arm, alongside minor abrasions on his legs, but no evident vascular or neurological injuries. Laboratory findings and a full-body CT scan revealed multiple fractures of the posterior vertebral arches and ribs on the left side, accompanied by subcutaneous emphysema and hemopneumothorax, as well as small bilateral pleural effusions. The scan also identified three distinct fractures of the left humerus: two on the shaft (one incomplete) and one supra-intercondylar fracture at the distal humerus with a comminuted intra-articular component, along with a radial neck fracture (Figure 1).

The hemopneumothorax was initially managed with a Bülau drain on the left side, and a long arm splint was applied, supported by a sling to stabilize the left arm.

Five days later, once the patient was deemed medically stable, he underwent surgery for definitive treatment of his fractures. The surgery began with the patient in a supine position, and his left arm positioned on an arm table. The proximal shaft fracture involved only one cortex and was managed conservatively. The central shaft fracture was addressed by utilizing an anterolateral approach; after open reduction, it was fixated with a seven-hole dynamic compression plate (DCP) 4.5 mm secured with three proximal and three distal screws. Subsequently, the patient was repositioned into lateral decubitus to address the supra-intercondylar fracture of the distal humerus, characterized as Gustilo I due to a wound below 1 cm. The ulnar nerve was carefully identified and protected throughout the osteotomy of the olecranon and during the handling of the peripheral comminuted fracture. The bone fragments were reduced and stabilized with Kirschner wires, followed by the application of a medial and a posterolateral plate in a perpendicular fashion (Medartis AG, Basel, Switzerland). The decision to fix the humerus in different planes (medial, lateral, and posterior) was made in order to minimize the risk of postoperative fractures between the plates and accommodate the use of longer plates as required.

**Figure 2:**
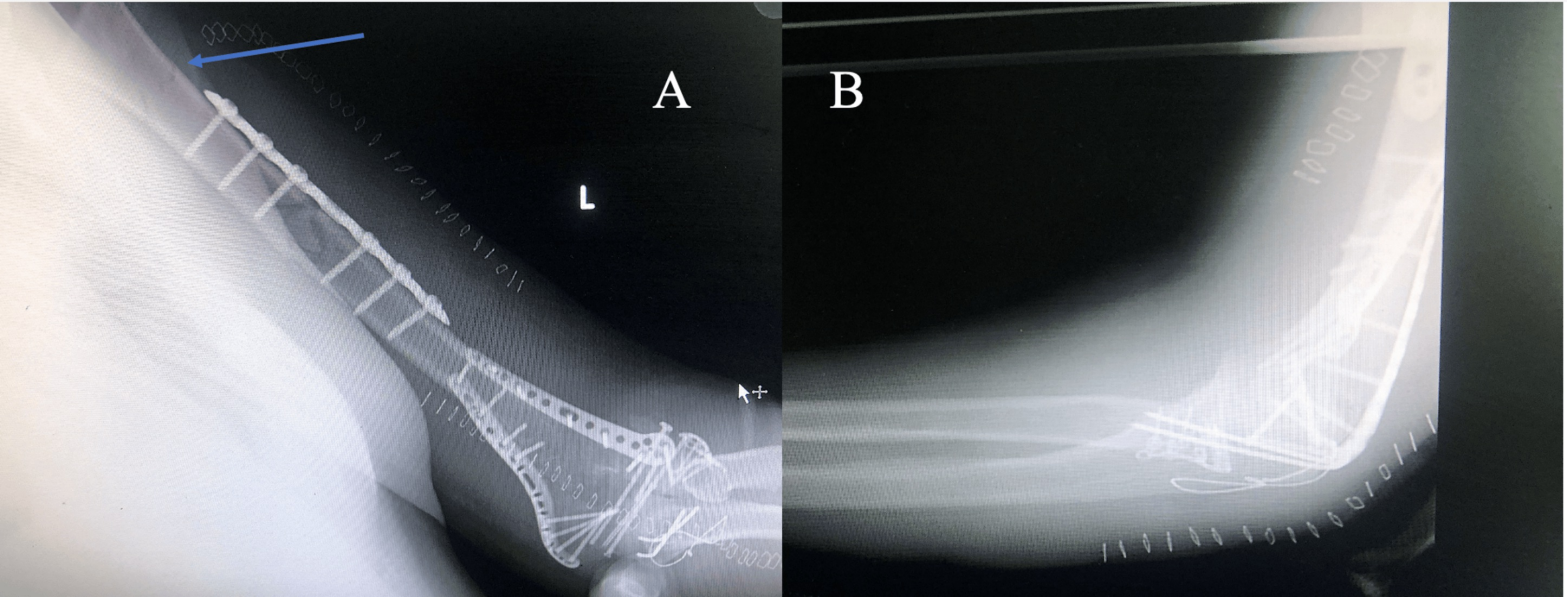
Immediate post-op anteroposterior (A) and lateral (B) radiographs of the left humerus and elbow. The untreated proximal humerus fracture is shown with the blue arrow.

The Boyd-Speed approach was employed to address the radial neck fracture; the anconeus muscle was transpositioned, and open reduction and internal fixation were performed with an anatomic radial neck plate and locking screws (Medartis AG, Basel, Switzerland). The surgery concluded with the fixation of the osteotomized olecranon using a tension band technique with two Kirschner wires and a figure of eight configured wires, and all operated sites were meticulously sutured in anatomical order. Postoperatively, the patient was transferred to the Intensive Care Unit (ICU) for resuscitation, with the elbow immobilized in a 90-degree flexion splint (Figure 2).

**Figure 3:**
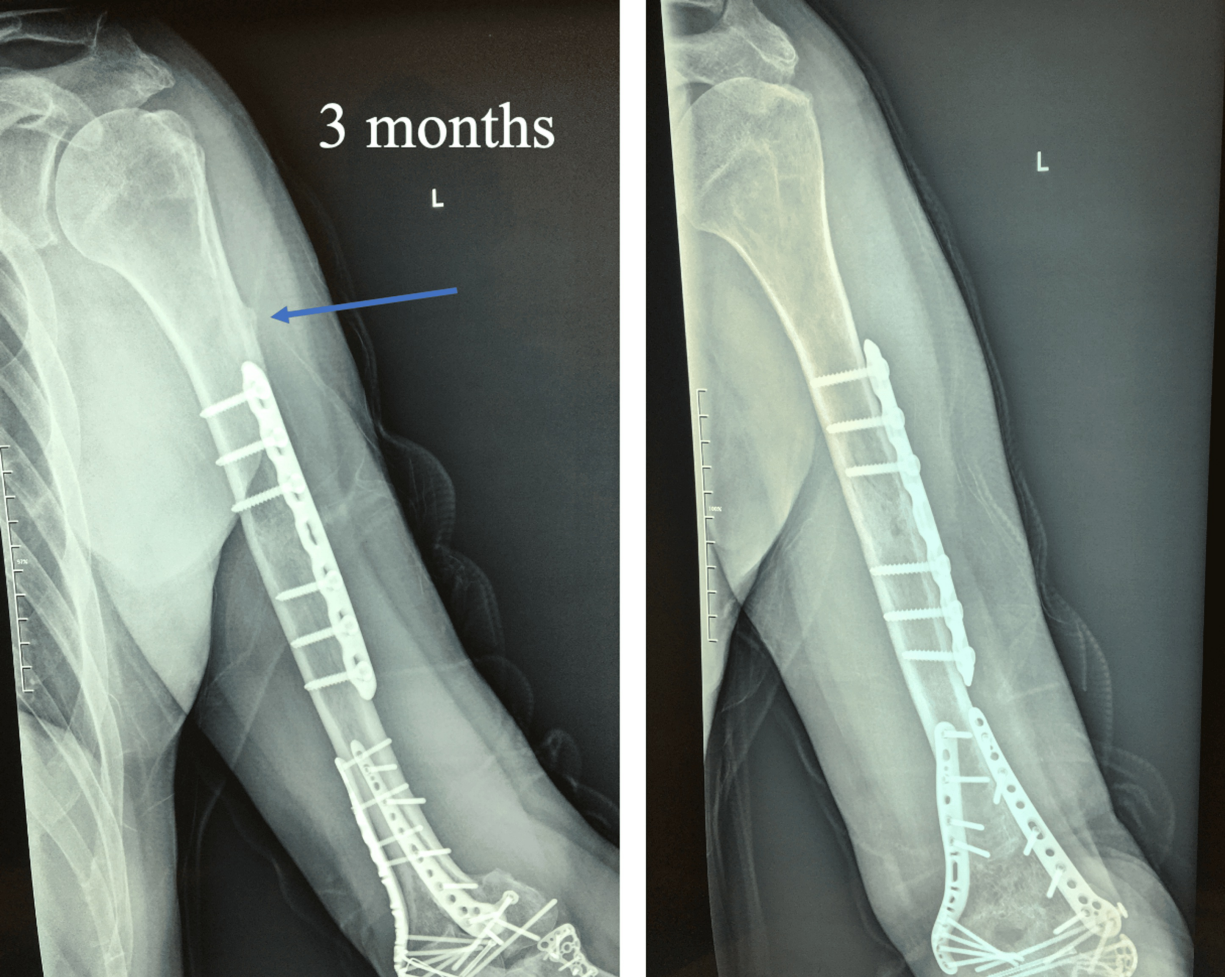
Radiographs at three months with the humerus in external rotation (left radiograph) and neutral position, in order to depict the fracture union. The union of the untreated proximal humerus is shown with the blue arrow.

This static splint was maintained for two weeks before being replaced by a dynamic splint, facilitating the gradual mobilization of the elbow over an additional four weeks. Three months post-operation, fracture union was noted, and the patient achieved a restored range of motion (ROM) from 25 to 130 degrees (Figure 3).

**Figure 4:**
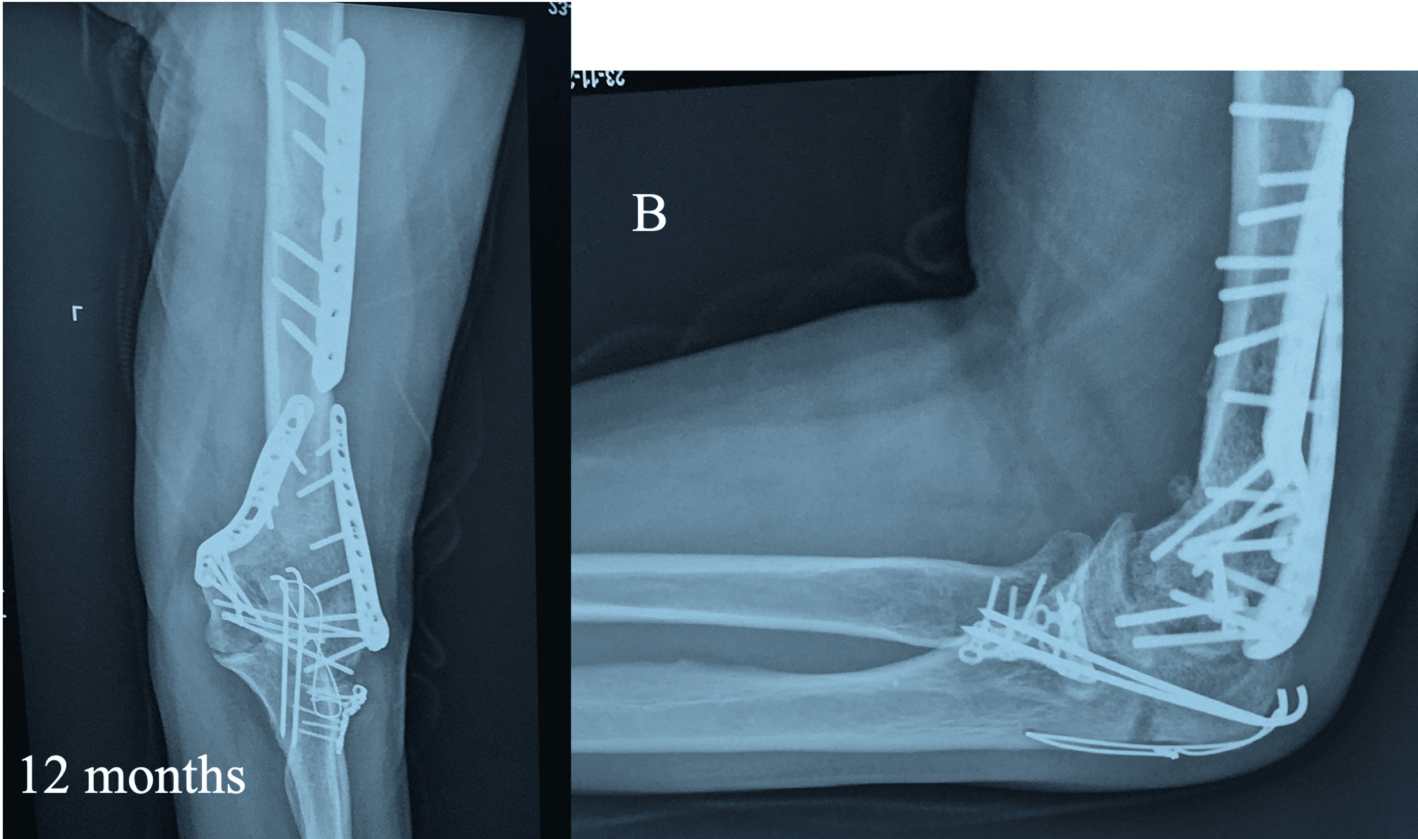
Radiographs at 12 months with the humerus in anteroposterior (left radiograph) and lateral positions in order to depict the union of all fractures.

 Radiographs at 12 months confirm the union of all fractures (Figure 4).

**Figure 5:**
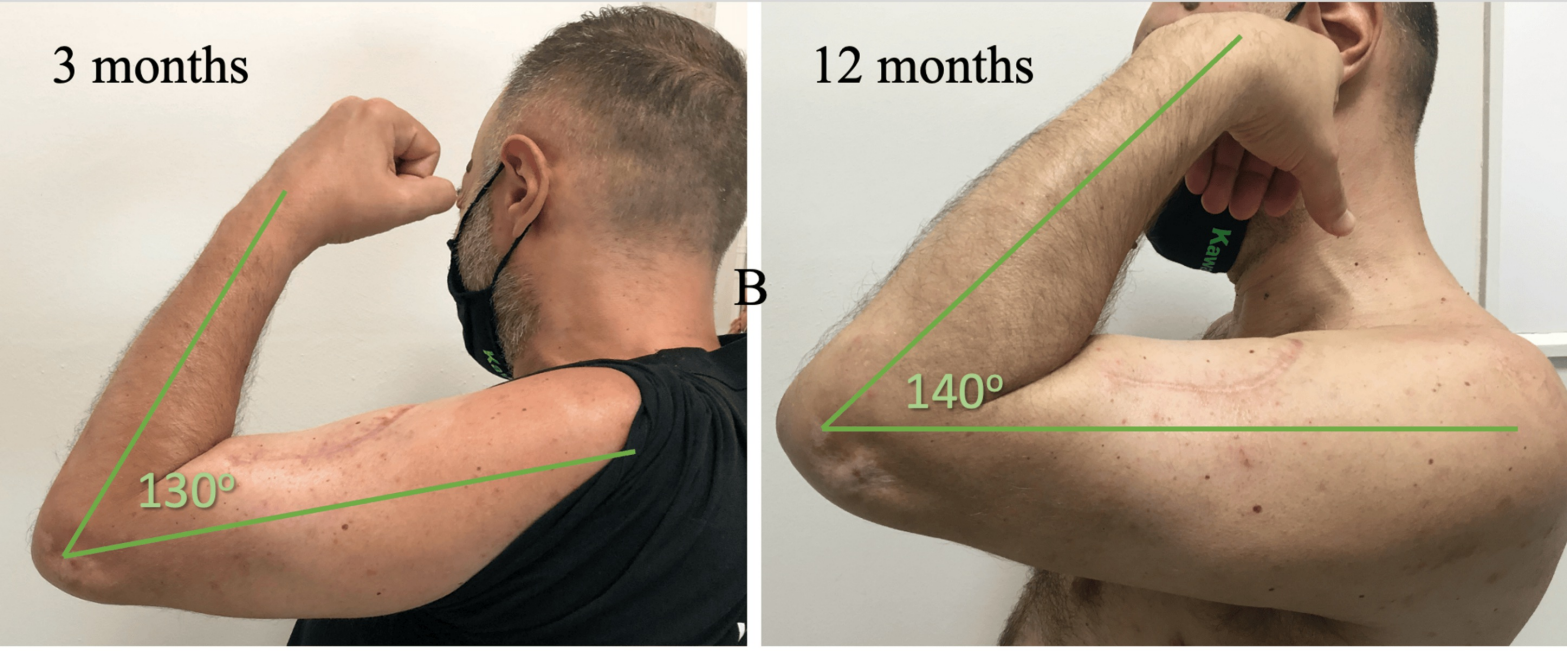
Patient's elbow in flexion at three and 12 months.

**Figure 6:**
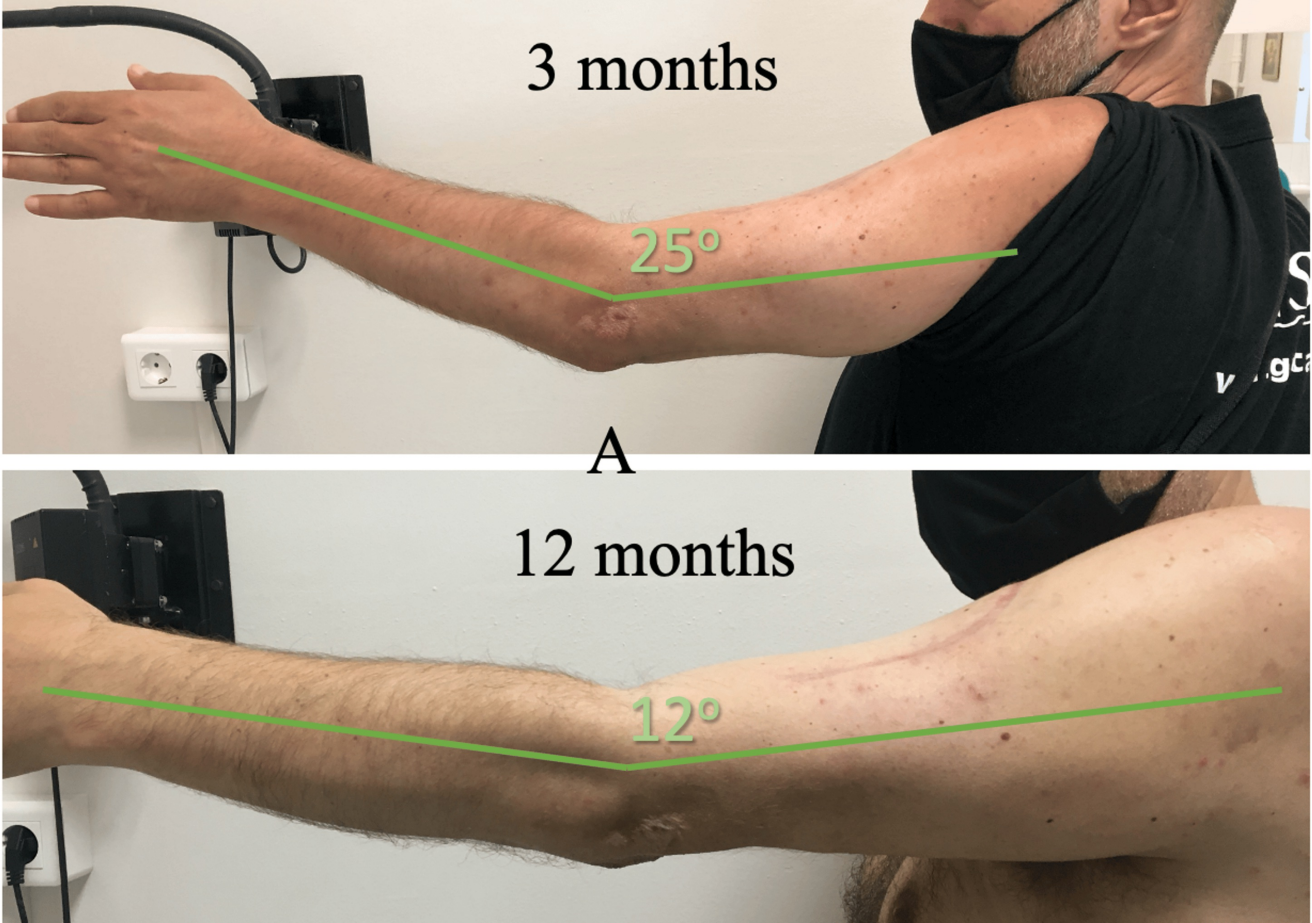
Patient's elbow in extension at three and 12 months.

At six months and a year later, his elbow ROM increased gradually as it reached a lack of 12° in extension and 140° of flexion (Figures 5, 6).

## Discussion

The management of complex humeral fractures, particularly those associated with the "floating elbow" phenomenon, necessitates an integrated approach that considers both the biomechanical challenges and the critical nature of maintaining neurovascular integrity. The "floating elbow" typically presents as a combination of ipsilateral humeral and forearm fractures, creating an unstable segment that significantly challenges both stability and function [5].

In this case, the decision to employ a dual approach for the humerus and use perpendicular plating posteriorly was influenced by the potential to improve biomechanical stability across the intricate multi-planar fracture interfaces, thereby effectively managing the inherent stresses associated with such injuries. Several studies support the use of perpendicular plating, indicating no significant difference in outcomes between perpendicular and parallel plating, thus affirming the efficacy of perpendicular plating in terms of elbow function and ROM post-recovery [6-10].

Preservation of neurovascular structures is crucial in surgeries involving the humerus, given the proximity of the radial nerve. Our meticulous surgical approach is aimed at minimizing the risk of iatrogenic damage and aligns with established practices for protecting nerve pathways during humeral fracture fixation [2].

The clinical outcomes observed in this case were encouraging, with the patient achieving a functional ROM and returning to daily activities. These results highlight the effectiveness of this plating arrangement in managing complex humeral fractures, as corroborated by studies that highlight its reliability in achieving anatomical restoration and mechanical stability necessary for early mobilization and rehabilitation [6,8,11].

## Conclusions

In conclusion, managing complex humeral fractures within the context of a "floating arm" injury variant requires a tailored approach that considers the anatomical challenges and specific biomechanical requirements of the injury. The application of plates in different planes in our case not only adheres to contemporary practices but also contributes to the broader discourse on optimizing treatment strategies for such high-energy trauma scenarios. Further research and clinical documentation are essential to refine these strategies and enhance recovery profiles for patients experiencing similar injuries.
